# PLAAT2 suppresses gastric cancer progression by facilitating cMyc ubiquitination and inhibiting MEK/ERK signaling

**DOI:** 10.1038/s41419-026-08546-y

**Published:** 2026-03-18

**Authors:** Mingfei Chu, Xialing Shi, Zhantai Shi, Yu Liang

**Affiliations:** 1 Department of Colorectal Surgery, Cancer Hospital of China Medical University, Cancer Hospital of Dalian University of Technology, Liaoning Cancer Hospital & Institute, Shenyang, China; 2 Department of Surgical Oncology and General Surgery, The First Hospital of China Medical University, Shenyang, China

**Keywords:** Gastric cancer, Ubiquitylation

## Abstract

Gastric cancer (GC) is a significant global public health issue due to its high incidence and limited therapeutic options. This study aimed to explore the role of phospholipase A and acyltransferase 2 (PLAAT2) in GC progression and its molecular mechanisms. A total of 116 pairs of GC and adjacent tissues, along with 116 paraffin-embedded GC tissue sections, were collected from the Cancer Hospital of China Medical University. The expression of PLAAT2 in GC tissues and cells was detected using quantitative reverse transcription–polymerase chain reaction and western blot assays. Its effects on proliferation, migration, invasion, and apoptosis were assessed using functional assays. The impact on mitogen-activated protein kinase (MEK)/extracellular signal-regulated kinase (ERK) signaling pathway and EMT-related proteins was examined through western blot. Immunoprecipitation–mass spectrometry (IP-MS), co-immunoprecipitation (co-IP), and ubiquitination assays were conducted to elucidate the molecular mechanisms of PLAAT2 to identify PLAAT2-interacting proteins, particularly its role in cMyc posttranslational regulation. In vivo xenograft models further validated the tumor-suppressive role of PLAAT2. We identified PLAAT2 as a differentially expressed gene associated with prognosis in the datasets of patients with GC. PLAAT2 was downregulated in GC and correlated with poor prognosis. Functional experiments demonstrated that PLAAT2 inhibited GC cell proliferation, migration, and invasion through the MEK/ERK signaling pathway. IP-MS and co-IP revealed that cMyc and tripartite motif containing 32 (TRIM32) were key PLAAT2-binding partners. PLAAT2 facilitated the recruitment of TRIM32 to promote cMyc ubiquitination and degradation, thereby suppressing the MEK/ERK signaling pathway and reducing oncogenic potential in vitro and in vivo. PLAAT2 functions as a tumor suppressor in GC by recruiting TRIM32 to facilitate cMyc ubiquitination and impair MEK/ERK-driven oncogenic signaling, highlighting the PLAAT2/TRIM32/cMyc axis as a potential therapeutic target.

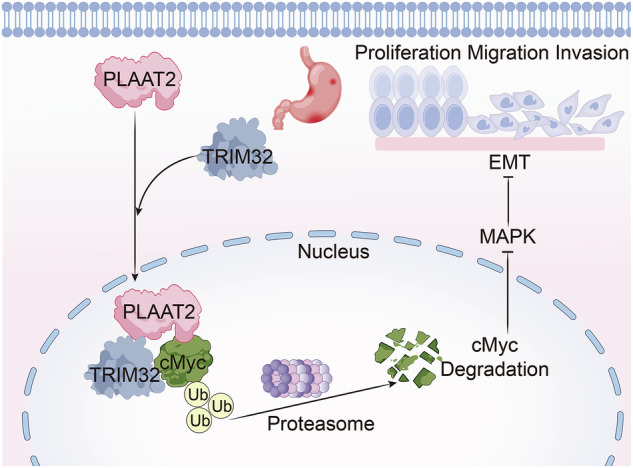

## Introduction

Gastric cancer (GC) is a prevalent malignancy with high incidence and mortality rates, posing a significant global public health challenge. An estimated one million new cases of GC are diagnosed annually, with more than 700,000 deaths, accounting for approximately 10% of global cancer-related mortality [1]. The development and progression of GC is a multifactorial process shaped by host genetics, infectious agents (*Helicobacter pylori* and Epstein-Barr virus), and dietary factors [2]. Recent years have witnessed an increase in GC incidence among younger individuals. Most cases are diagnosed in advanced stages due to the vague early symptoms of GC and limited screening uptake, leading to a poor prognosis [3]. Identifying potential biomarkers is essential for elucidating the complex mechanisms driving GC progression and enhancing patient prognosis.

Phospholipase A and acyltransferase 2 (PLAAT2) was initially identified in the SW480 colon cancer cell line. It is located on human chromosome 11, encoding 162 amino acids. Emerging studies have highlighted its involvement in cancer, particularly in RAS-mediated cellular transformation [4]. Transfection of PLAAT2 into HCT116, HeLa, and MCF-7 cells inhibits colony formation in these cancer cell lines [5]. Furthermore, PLAAT2 overexpression suppresses the activation of both wild-type and mutant RAS in HtTA cervical cancer cells, leading to enhanced cell death. These effects depend on the C-terminal hydrophobic structure, and the truncation of the last 26 amino acids of the C-terminus abolishes the pro-apoptotic, anti-RAS, and growth-inhibitory activities of PLAAT2 [6]. To date, the function of PLAAT2 in GC has not been explored. Also, whether PLAAT2 can serve as a potential diagnostic and therapeutic target for GC requires investigation.

Myelocytomatosis viral oncogene (MYC) is located on chromosome 8q24 and encoded by a gene comprising three exons, which can produce multiple MYC transcript variants through alternative splicing [7]. The MYC gene family includes three members, cMyc, lMyc, and nMyc, all of which are basic helix–loop–helix leucine zipper DNA-binding proteins [8]. CMyc, a key proto-oncogene in the MYC family, is dysregulated in more than half of all malignancies and plays a crucial role in cell cycle regulation, apoptosis, proliferation, as well as cell migration and invasion [9]. The MYC protein has a short half-life of approximately 30 min and undergoes degradation primarily through the ubiquitin–proteasome pathway [10]. cMyc plays a significant role in osteosarcoma by activating the MEK/ERK pathway. Its overexpression has been shown to enhance the malignant abilities of MG63 and SAOS2 cells, accompanied by the upregulation of phosphorylated extracellular signal-regulated kinase (p-ERK), phosphorylated mitogen-activated protein kinase (p-MEK), matrix metalloproteinase-2 (MMP-2), and MMP-9. Treatment with the MEK inhibitor PD98059 suppresses cell invasion and downregulates MMP-2 and MMP-9 expression [11]. cMyc functions as a transcription factor in lung cancer to promote the expression of LINC01503, thereby activating the ERK/mitogen-activated protein kinase (MAPK) signaling pathway to enhance proliferation, migration, and invasion of lung cancer while concurrently suppressing apoptosis [12]. The MAPK pathway is a crucial intracellular signaling cascade regulating various cellular processes, including differentiation, proliferation, apoptosis, and migration and invasion [13]. The MAPK pathway is activated by various extracellular signals, including growth factors, cytokines, and stress stimuli. Extracellular ligands bind to receptors (e.g., receptor tyrosine kinases), inducing conformational changes in the receptors. The activated receptors initiate downstream protein kinases, which then trigger the phosphorylation of substrates, leading to changes in gene expression and ultimately influencing cellular processes [14, 15]. The RAS/RAF/MEK/ERK pathway, a classic MAPK pathway, is widely involved in tumor cell proliferation, apoptosis, and migration and invasion [16].

The biological role and underlying molecular mechanisms of PLAAT2 remain poorly understood. This study revealed that PLAAT2 expression was significantly downregulated in GC and associated with poor patient prognosis. Mechanistic studies revealed that PLAAT2 interacted with cMyc and downregulated its expression. PLAAT2 facilitated the ubiquitin-mediated degradation of cMyc by recruiting a tripartite motif containing 32 (TRIM32), thereby inhibiting the MEK/ERK signaling pathway and ultimately suppressing GC proliferation and metastasis. In summary, we investigated the impact of PLAAT2 on GC progression and its underlying molecular mechanisms. These findings establish a theoretical foundation of PLAAT2, highlighting its role in tumor suppression.

## Materials and methods

### Identification of differentially expressed genes

The expression and clinical data of GC were downloaded from The Cancer Genome Atlas (TCGA) and processed using Perl software, converting Ensembl IDs into gene names to generate sample files. The R package “DESeq2” was used to screen for DEGs, using the criteria |log2 fold change | >1 and false discovery rate <0.05. Subsequently, we extracted the clinical data of GC and performed prognostic analysis using the “Survival” and “Survminer” packages to identify DEGs associated with GC prognosis. We identified and analyzed methylation-regulated differentially expressed genes following the protocol used in previous studies [17].

### Tissue sample collection and cell culture

A total of 116 pairs of GC and adjacent tissues, along with 116 paraffin-embedded GC tissue sections, were collected from the Cancer Hospital of China Medical University. The inclusion criteria were complete clinicopathological data and follow-up information of patients with GC after surgery. The study was approved by the Ethics Committee of the Cancer Hospital of China Medical University.

Five GC cell lines (AGS, HGC27, MKN28, MGC803, and MKN45) and one gastric mucosal epithelial cell line GES1 were used in this study. The cell lines were obtained from the Shanghai Cell Bank of the Chinese Academy of Sciences. The AGS cell line was maintained in Ham’s F-12 medium (Corning, NY, USA). In contrast, the remaining cell lines were cultured in the RPMI-1640 medium (Gibco, MA, USA), both supplemented with 10% fetal bovine serum (Gibco) and 1% penicillin–streptomycin to prepare complete culture media.

### Cell transfection

The log-phase cells (1 × 10⁵/well) were seeded in a six-well plate and cultured for 24 h. For shRNA transfection, Lipofectamine 3000 (Invitrogen, Thermo Fisher Scientific, MA, USA) and shRNA (GenePharma, Jiangsu, China) were mixed in a serum-free medium, incubated for some time, and added to cells. For lentiviral transfection, the cells were seeded a day prior to infection (30%–50% confluence). Lentivirus (GenePharma) and Polybrene (Sigma–Aldrich, MO, USA) were added, incubated for 16 h, and replaced with a fresh complete medium. After 72 h, puromycin (Beyotime, Shanghai, China) selection was used to obtain stable clones. The sequences of shRNAs are shown in Supplementary Table 1.

### Quantitative reverse transcription–polymerase chain reaction and western blot analysis

The primers used in this study are listed in Supplementary Table 2. For specific experimental methods and reagents, refer to a previous study [18]. Moreover, we treated cells with different concentrations of 5-Aza-CdR (a DNA methyltransferase inhibitor), extracted proteins after 48 h, and assessed PLAAT2 expression by western blot analysis. The antibodies are shown in the Supplementary Material (Reagents and antibodies).

### Immunohistochemistry

The tissues were fixed, paraffin-embedded, and sectioned (4 μm) using a microtome (Leica, Wetzlar, Germany). After dewaxing, rehydration, and antigen retrieval in citrate buffer (Beyotime), the sections were blocked with 3% hydrogen peroxide (Solarbio) and a nonspecific blocker (Solarbio) and then incubated with the anti-PLAAT2 (1:200, Thermo Fisher Scientific) at 4 °C overnight. After washing with phosphate-buffered saline (PBS; Gibco), a biotin-labeled secondary antibody (ZSGB-BIO, Beijing, China) and streptavidin–biotin complex (SABC; ZSGB-BIO) were used sequentially. 3,3’-Diaminobenzidine (Beyotime) was used for chromogenic detection (5–10 min), followed by hematoxylin counterstaining (Beyotime), dehydration, and mounting with neutral resin (Solarbio). The staining intensity and percentage of positive cells were evaluated under a microscope. The scoring criteria were as follows: (1) staining intensity: 0 = negative (no staining); 1 = low expression (light yellow); 2 = moderate expression (yellow); and 3 = high expression (brown-yellow); and (2) percentage of positive cells: 0 = 0%–10%; 1 = 11%–25%; 2 = 26%–50%; 3 = 51%–75%; and 4 ≥ 75%.

### Cell proliferation assay

The cell proliferation ability was assessed by Cell Counting Kit-8 (CCK-8; Solarbio) and colony formation assay. The operating steps and reagents were followed from a previous study [18].

### Migration and invasion assay

We used Matrigel (Corning) and Transwell nesting (Corning) to perform the migration and invasion assays, respectively. The specific experimental details were the same as in a previous study [18].

### Cell apoptosis assay

The log-phase cells were transfected at 40%–60% confluence, and apoptosis was assessed after 48 h. The cells were washed with PBS, digested with EDTA-free trypsin (Beyotime), and centrifuged. The supernatant was discarded, the cells were resuspended in 1 mL of PBS and counted, and 2 × 10⁵ cells were collected. After centrifugation, Annexin V–FITC/PI staining solution (Beyotime) was added, and the cells were incubated for 15 min. Apoptosis was analyzed using a flow cytometer (BD Biosciences, USA), and the apoptosis rate was calculated.

### Immunoprecipitation–mass spectrometry

The AGS cells were prepared for analysis by adding an appropriate amount of IP-grade lysis buffer (Beyotime) to the cells, and the cells were incubated for complete lysis. The lysate was centrifuged, and the supernatant was transferred to a new EP tube. Protein A/G Magnetic Beads (Thermo Fisher Scientific) were added, and a magnetic separator was used to remove the supernatant. The beads were washed three times with a wash buffer (Beyotime). Subsequently, 4 μg of antibody was added, and the tube was incubated overnight at 4 °C on a rotating mixer. The next day, the beads were collected using the magnetic separator, and the supernatant was discarded. Next, 3 mg of protein lysate was added and incubated overnight at 4 °C. After washing, 1× loading buffer (Beyotime) was added, mixed, and heated at 100 °C for 10 min to elute the proteins. The samples were then sent for mass spectrometry analysis.

### Co-immunoprecipitation

The log-phase cells were lysed with a non-denaturing lysis buffer (Beyotime) at 4 °C for 30 min, and the supernatant was collected after centrifugation. Protein A/G Magnetic Beads (Thermo) were added, magnetically separated, and incubated with 2 μg of antibody at 4 °C overnight. Subsequently, 10% of the lysate was collected as the Input group, whereas the remaining sample was incubated overnight. The beads were washed, magnetically separated, mixed with 1× loading buffer (Beyotime), heated at 100 °C for 10 min, and centrifuged. The supernatant was used for western blot analysis.

### Immunofluorescence

The log-phase cells were digested, centrifuged, and resuspended. Approximately 5000 cells were seeded onto coverslips in a six-well plate, followed by 24-h incubation. The cells were fixed with 4% paraformaldehyde (Solarbio), permeabilized with 0.5% Triton X-100 (Beyotime), and blocked with BSA (Beyotime). They were incubated first with the primary antibody (Abcam) at 4 °C overnight and then with the fluorescent secondary antibody (Solarbio) for 2 h in the dark. DAPI (Beyotime) was used for nuclear staining, followed by an antifade mounting medium (Solarbio). The images were captured using a confocal microscope.

### Methylation-specific PCR (MSP)

Genomic DNA was extracted from cell samples using a cell DNA extraction kit (Abcam), and bisulfite conversion of the genomic DNA was performed using the EpiTect Rapid DNA Bisulfite Kit (QIAGEN). The PCR reaction system was then prepared following the instructions for the Go Taq Green Master Mix (Promega). A 5-μL aliquot of the amplification product was mixed with 1 μL of 5× loading buffer, pipetted until thoroughly homogenized, and loaded into a 2% agarose gel for electrophoresis. The imaging results were observed and analyzed using a gel electrophoresis imaging analysis system (Thermo). Primer sequences are provided in Supplementary Table 4.

### Xenograft tumor formation in nude mice

A total of 24 nude mice were randomly assigned to 6 groups. The log-phase cells were transfected according to the experimental groups, digested, centrifuged, and resuspended. Approximately, 2 × 10^6^ cells per group were counted and prepared. The cells were injected subcutaneously into the posterior axillary region of the mice, which had rich blood supply. The tumor growth and mouse health were monitored every 3 days, and the tumor size was measured using a caliper, recording the maximum length and width. The mice were weighed after approximately 4 weeks, when tumors reached a suitable size, and euthanized. Their tumors were excised, weighed, and photographed.

### Statistical analysis

All statistical analyses were performed using SPSS 19.0 and GraphPad Prism 9.0 software. Before statistical analysis, the variability of each group of data and the assumptions of the tests were checked. Kaplan–Meier curves and log-rank tests were used to evaluate overall survival between different groups. Paired samples were analyzed using the *t* test, whereas one-way analysis of variance was used for comparisons among multiple groups. The chi-square test was used to assess correlations between categorical variables (^*^P < 0.05, ^**^P < 0.01). All experiments were independently repeated three times.

## Results

### PLAAT2 was downregulated in GC tissues and cells

**Fig. 1:**
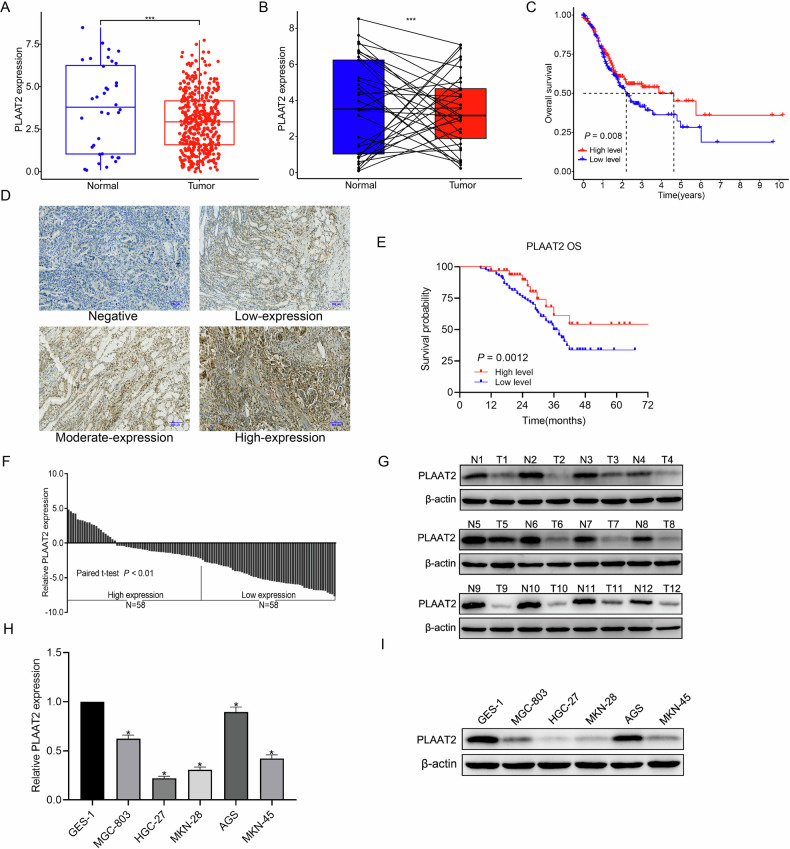
PLAAT2 was downregulated in gastric cancer tissues and cells. **A, B** Analysis of PLAAT2 expression in gastric cancer and normal tissues using the TCGA database. **C** Kaplan–Meier survival analysis exploring the relationship between PLAAT2 expression and prognosis in gastric cancer. **D, E** Immunohistochemical analysis of PLAAT2 expression in gastric cancer tissues and adjacent normal tissues, and its correlation with patient prognosis. **F, G** Quantitative real-time PCR (qRT-PCR) and western blot analysis of PLAAT2 expression in gastric cancer tissues and adjacent normal tissues. **H, I** qRT-PCR and western blot analysis of PLAAT2 expression in normal gastric mucosal epithelial cells and gastric cancer cells. ^*^P < 0.05, ^**^P < 0.01.

We downloaded transcriptomic data from the TCGA database, including 32 adjacent noncancerous samples and 375 GC samples. Differential gene expression analysis revealed that PLAAT2 expression was significantly lower in GC tissues compared with adjacent normal tissues (Fig. 1, 1B). We then analyzed the correlation between PLAAT2 expression and survival outcomes in 375 patients with GC. Patients with low PLAAT2 expression exhibited significantly poor overall survival (Fig. 1C). Immunohistochemical experiments confirmed that PLAAT2 expression was lower in GC tissues than in adjacent normal tissues, and low PLAAT2 expression was significantly correlated with worse overall survival (Fig. 1D, E). Then, we analyzed the correlation between PLAAT2 expression levels and clinicopathological parameters of patients using the chi-square test. The results revealed that patients in the PLAAT2 low-expression group had a higher T stage (*P* = 0.013), a higher incidence of lymph node metastasis (*P* = 0.020), and a more advanced TNM stage (*P* = 0.005) (Supplementary Table 3). Furthermore, quantitative reverse transcription–polymerase chain reaction (qRT-PCR) and western blot results indicated that PLAAT2 expression was significantly reduced in GC tissues compared with adjacent noncancerous tissues (Fig. 1F, G). Additionally, qRT-PCR and western blot analyses revealed that PLAAT2 was downregulated in GC cell lines (Fig. 1H, I). Additionally, we further explored the mechanism underlying the downregulation of PLAAT2 in GC. In our preliminary research, we found that the upstream promoter region of PLAAT2 was significantly enriched with CpG islands and exhibited high levels of methylation in GC tissues (Supplementary Fig. 1A, B). Correlation analysis revealed a negative association between PLAAT2 expression and the methylation level of its promoter region (Supplementary Fig. 1C). Kaplan–Meier survival curves demonstrated that hypermethylation of the PLAAT2 promoter was significantly associated with poorer prognosis in patients with GC (Supplementary Fig. 1D). Subsequently, we assessed the methylation status of the CpG islands in the PLAAT2 gene promoter region using MSP. The MSP results showed hypomethylation of the PLAAT2 gene promoter region in the normal gastric mucosal epithelial cell line GES-1, whereas hypermethylation was observed in five GC cell lines (Supplementary Fig. 1E). To assess whether PLAAT2 downregulation was driven by promoter hypermethylation, we treated HGC-27 and MKN-28 GC cell lines, both of which exhibited relatively low PLAAT2 expression, with the demethylating agent 5-Aza-CdR. Western blot analysis revealed upregulation of PLAAT2 protein expression after treatment with 5-Aza-CdR, showing a clear dose-dependent relationship with drug concentration (Supplementary Fig. 1F). These results indicated that PLAAT2 expression was regulated by DNA methylation.

### Knockdown of PLAAT2 promoted progression of GC

**Fig. 2:**
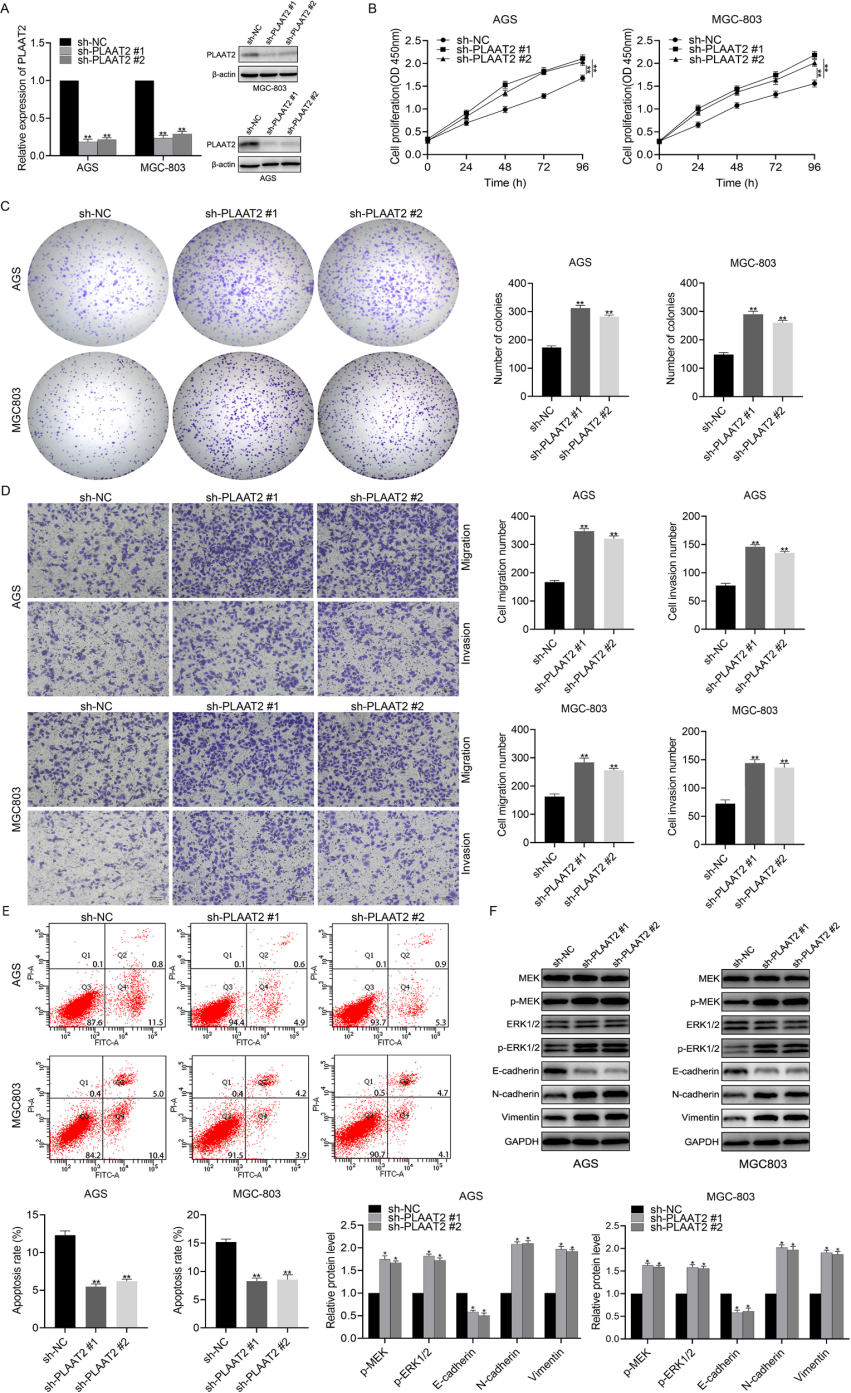
Knockdown of PLAAT2 promoted proliferation, migration, and invasion of gastric cancer cells. **A** qRT-PCR and western blot analysis of PLAAT2 expression in AGS and MGC803 cells transfected with sh-NC and sh-PLAAT2. **B** CCK-8 assay evaluating the effect of PLAAT2 knockdown on the proliferation of AGS and MGC803 cells. **C** Colony formation assay analyzing the impact of PLAAT2 knockdown on the clonogenic ability of AGS and MGC803 cells. **D** Transwell assay assessing changes in migration and invasion abilities of AGS and MGC803 cells after PLAAT2 knockdown. **E** Apoptosis assay analyzing the effect of PLAAT2 knockdown on apoptosis levels in AGS and MGC803 cells. **F** Western blot analysis of changes in the MEK/ERK pathway and EMT-related proteins after PLAAT2 knockdown. ^**^P < 0.01.

AGS and MGC803 cell lines, exhibiting relatively high expression of PLAAT2 in knockdown experiments, were selected to further reveal the functional role of PLAAT2 in GC. Following transfection with sh-PLAAT2#1 and sh-PLAAT2#2, the knockdown efficiency was confirmed through western blot and qRT-PCR (Fig. 2A). The CCK-8 assay showed that PLAAT2 knockdown significantly enhanced the proliferation of AGS and MGC803 cell lines, as indicated by absorbance measurements after 0, 24, 48, 72, and 96 h (Fig. 2B). Additionally, PLAAT2 knockdown significantly increased the colony-forming ability of AGS and MGC803 cell lines (Fig. 2C). Transwell assays indicated that PLAAT2 knockdown enhanced the migration and invasion capabilities of AGS and MGC803 cells (Fig. 2D). Annexin V–FITC/PI double staining showed that PLAAT2 knockdown significantly reduced the apoptosis rate of AGS and MGC803 cells compared with that in the control group (Fig. 2E). GSEA of PLAAT2 revealed significant enrichment in pathways related to the MEK/ERK signaling pathway and epithelial–mesenchymal transition (EMT), indicating the role of PLAAT2 in these processes (Supplementary Fig. 2). We further explored the correlation between PLAAT2 expression, MEK/ERK pathway activation, and EMT regulation in GC. Western blot analysis demonstrated that PLAAT2 knockdown in AGS and MGC803 cells did not affect total MEK and ERK1/2 protein levels; however, it significantly upregulated phosphorylated MEK and ERK1/2. Additionally, EMT-related markers showed a decrease in E-cadherin expression, accompanied by increased N-cadherin and Vimentin expression, indicating enhanced EMT progression (Fig. 2F).

### Overexpression of PLAAT2 inhibited the progression of GC

**Fig. 3:**
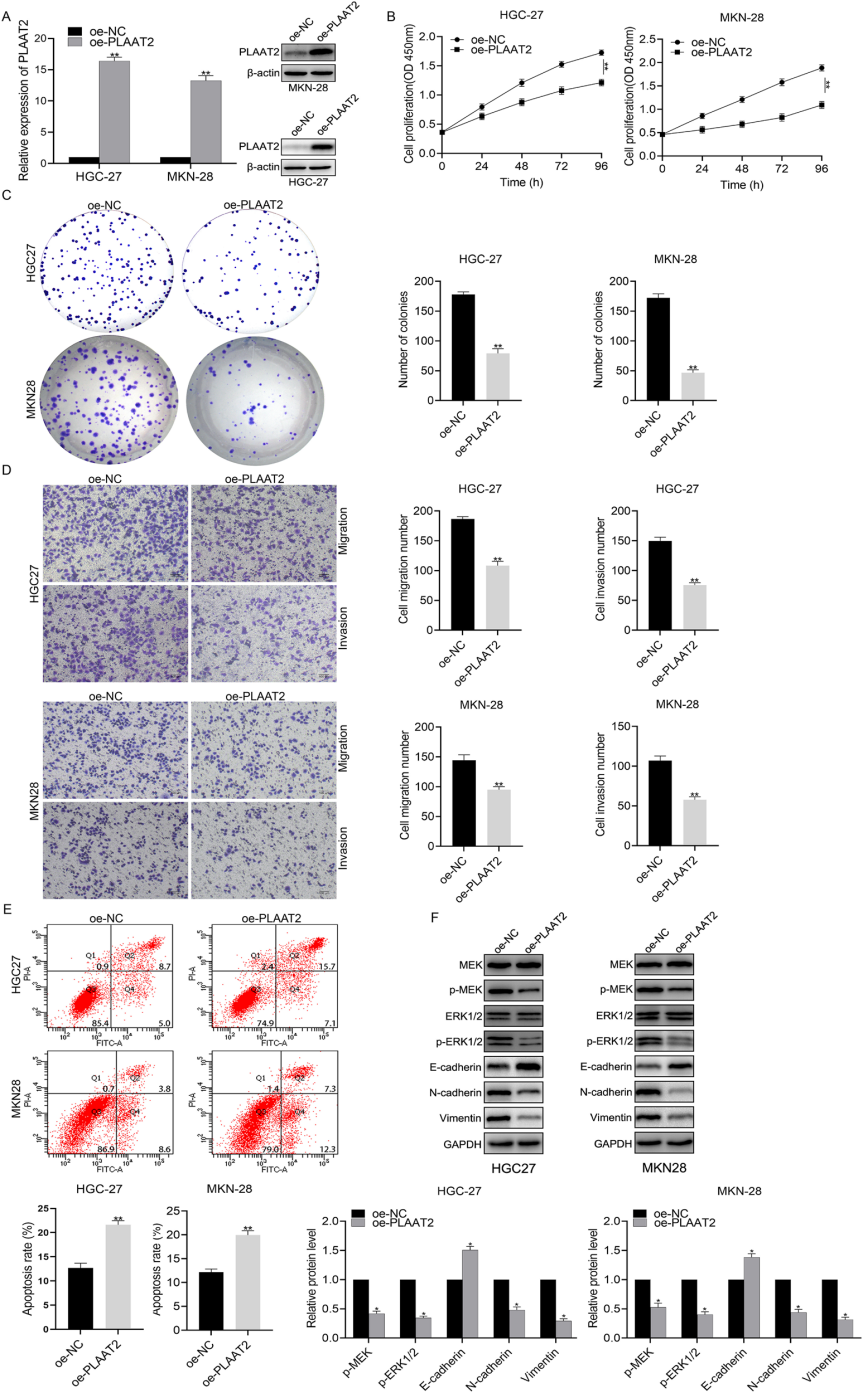
PLAAT2 Overexpression inhibited the proliferation, migration, and invasion of gastric cancer cells. **A** qRT-PCR and western blot analysis of PLAAT2 expression in HGC27 and MKN28 cells transfected with oe-NC and oe-PLAAT2. **B** CCK-8 assay evaluating the effect of PLAAT2 overexpression on the proliferation of HGC27 and MKN28 cells. **C** Colony formation assay analyzing the impact of PLAAT2 overexpression on the clonogenic ability of HGC27 and MKN28 cells. **D** Transwell assay assessing changes in migration and invasion abilities of HGC27 and MKN28 cells after PLAAT2 overexpression. **E** Apoptosis assay analyzing the effect of PLAAT2 overexpression on apoptosis levels in HGC27 and MKN28 cells. **F** Western blot analysis of changes in the MEK/ERK pathway and EMT-related proteins after PLAAT2 overexpression. ^**^P < 0.01.

HGC27 and MKN28 cells, exhibiting low PLAAT2 expression, were selected for overexpression experiments. Following lentiviral transfection, successful PLAAT2 overexpression was confirmed (Fig. 3A). CCK-8 and colony formation assays revealed that PLAAT2 overexpression significantly reduced the proliferation of HGC27 and MKN28 cells (Fig. 3B, C). Transwell assays further demonstrated that PLAAT2 overexpression reduced the migration capabilities of HGC27 and MKN28 cell lines (Fig. 3D). Apoptosis assays showed that PLAAT2 overexpression significantly increased the apoptosis rate of HGC27 and MKN28 cells (Fig. 3E). Western blot analysis revealed that PLAAT2 overexpression did not alter total MEK and ERK1/2 protein levels but significantly downregulated phosphorylated MEK and ERK1/2 and also inhibited EMT (Fig. 3F).

### PLAAT2 downregulated cMyc expression by promoting its ubiquitination and degradation

**Fig. 4:**
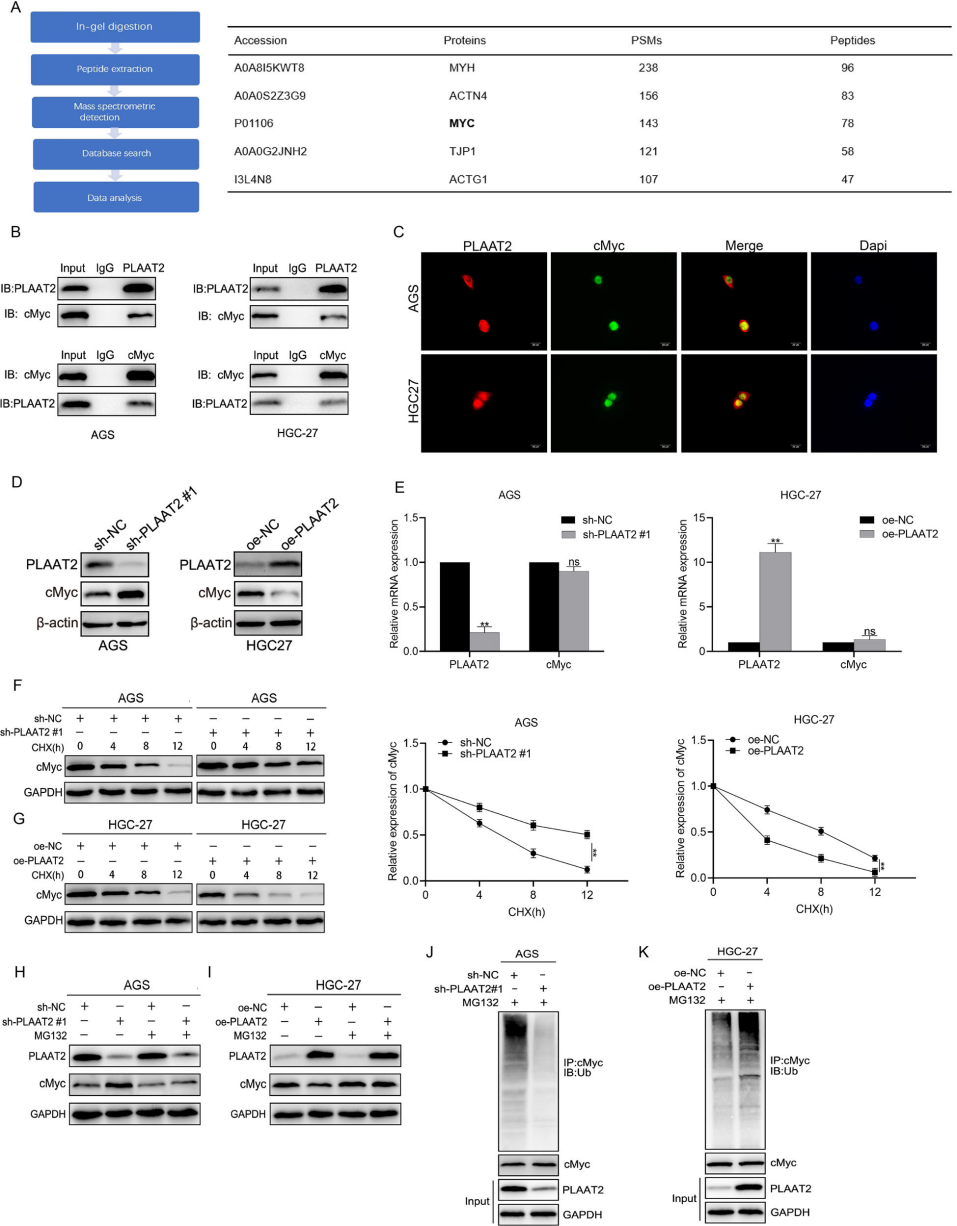
PLAAT2 downregulated cMyc expression by promoting its ubiquitination and degradation. **A** IP-MS screening of the top-five proteins interacting with PLAAT2. **B** Co-immunoprecipitation (Co-IP) confirming the interaction between PLAAT2 and cMyc. **C** Immunofluorescence showing co-localization of PLAAT2 and cMyc. **D, E** Western blot and qRT-PCR analysis of cMyc expression after knockdown or overexpression of PLAAT2. **F, G** Protein half-life assay evaluating the effect of PLAAT2 knockdown or overexpression on cMyc protein stability. **H, I** MG132 inhibited PLAAT2-mediated ubiquitination and degradation of cMyc. **J, K** Co-IP assay detecting the effect of PLAAT2 knockdown or overexpression on cMyc ubiquitination levels. ^**^P < 0.01, ns means no significance.

We identified 233 potential PLAAT2-interacting proteins using immunoprecipitation–mass spectrometry (IP-MS), with MYC, encoding the transcription factor cMyc, being particularly notable (Fig. 4A). MYC proto-oncogenes encode transcription factors such as cMyc, nMyc, and lMyc, which are frequently activated in cancers. Among these, cMyc plays a crucial role in cell proliferation, apoptosis, and metabolism. It has been validated to activate the MEK/ERK signaling pathway in osteosarcoma and lung cancer, promoting tumor invasion and metastasis. Therefore, we selected cMyc for further investigation. Co-immunoprecipitation (Co-IP) assays confirmed the direct interaction between PLAAT2 and cMyc (Fig. 4B), whereas immunofluorescence (IF) analysis demonstrated their co-localization (Fig. 4C), further supporting the functional relevance of this interaction in GC. Western blot analysis showed that PLAAT2 knockdown increased cMyc expression, whereas PLAAT2 overexpression decreased cMyc expression (Fig. 4D). QRT-PCR results indicated that PLAAT2 did not regulate cMyc at the mRNA level (Fig. 4E). Therefore, we hypothesized that PLAAT2 regulated cMyc expression through ubiquitination. Cycloheximide (CHX) chase assays demonstrated that PLAAT2 knockdown significantly delayed cMyc degradation, whereas PLAAT2 overexpression markedly accelerated it, suggesting its role in cMyc stability regulation (Fig. 4F, 4, G). MG132 treatment reversed the effects of PLAAT2 knockdown or overexpression on cMyc expression (Fig. 4H, I). Co-IP experiments confirmed that PLAAT2 knockdown reduced cMyc ubiquitination, whereas PLAAT2 overexpression increased it (Fig. 4J, K).

### PLAAT2 promoted cMyc ubiquitination and degradation by recruiting TRIM32

**Fig. 5:**
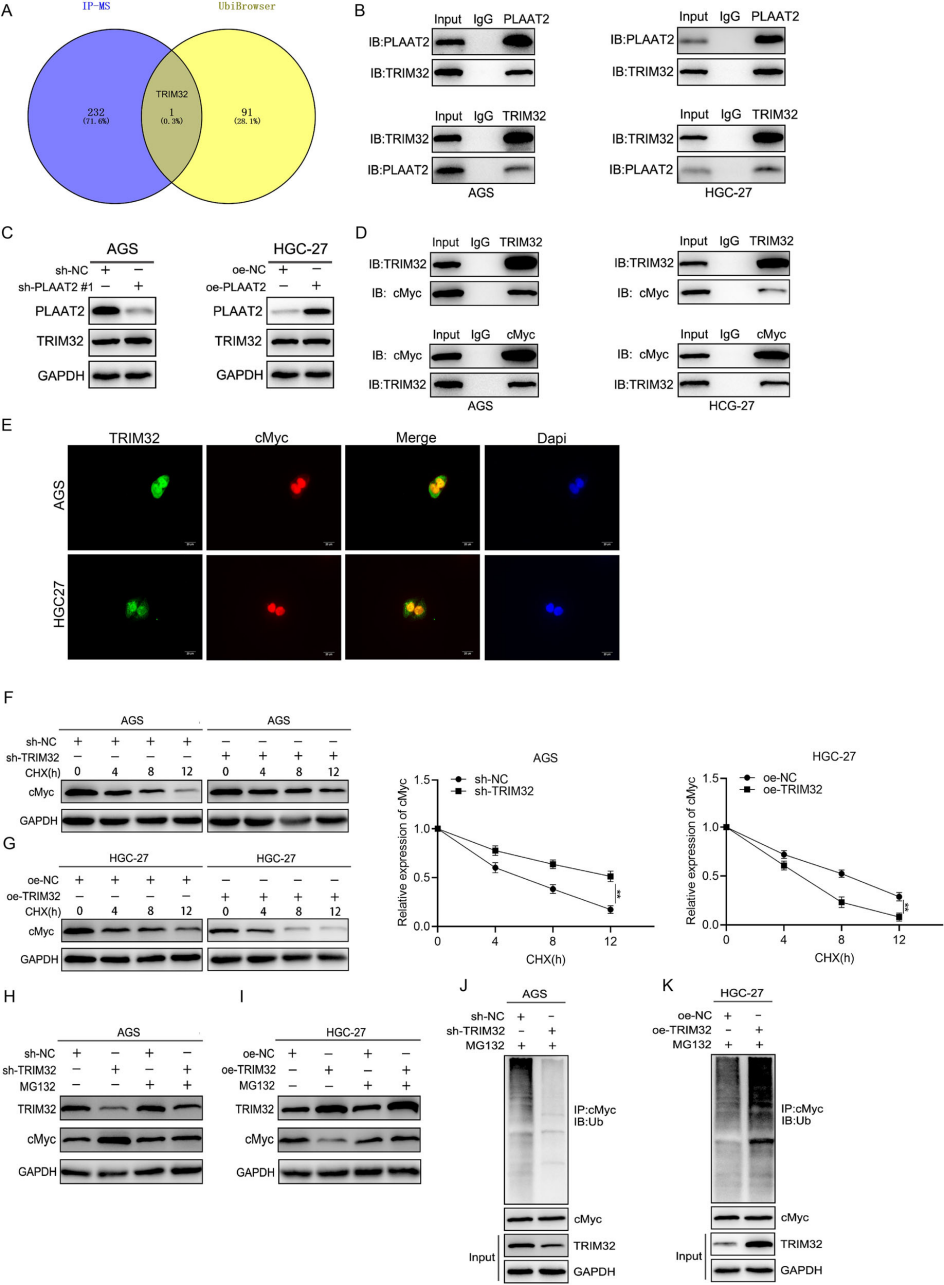
PLAAT2 promoted cMyc ubiquitination and degradation by recruiting TRIM32. **A** UbiBrowser database and IP-MS results identifying TRIM32 as the E3 ubiquitin ligase mediating PLAAT2-induced cMyc ubiquitination. **B** Co-IP confirming the interaction between PLAAT2 and TRIM32. **C** Western blot analysis of TRIM32 expression after PLAAT2 knockdown or overexpression. **D** Co-IP confirming the interaction between TRIM32 and cMyc. **E** Immunofluorescence showing co-localization of TRIM32 and cMyc. **F**, **G** Cycloheximide (CHX) assay evaluating the effect of TRIM32 on cMyc protein half-life. **H**, **I** MG132 reversed the effect of TRIM32 knockdown or overexpression on cMyc expression. **J**, **K** Co-IP assay detecting the effect of TRIM32 on cMyc ubiquitination levels. ^**^P < 0.01.

Previous studies demonstrated that PLAAT2 promoted cMyc ubiquitination and degradation. However, research indicates that PLAAT2 does not function as an E3 ubiquitin ligase, and structural analysis reveals that PLAAT2 lacks the catalytic domains necessary for E3 ligase activity. Therefore, the mechanism by which PLAAT2 mediates cMyc ubiquitination requires further investigation. We hypothesized that PLAAT2 may regulate cMyc ubiquitination by interacting with and recruiting an E3 ubiquitin ligase. We used the UbiBrowser database (http://ubibrowser.bio-it.cn/ubibrowser_v3/) to predict 92 potential E3 ligases that might mediate cMyc ubiquitination. We intersected these candidates with the 233 PLAAT2-interacting proteins identified through IP-MS, identifying TRIM32 as a promising candidate (Fig. 5A). TRIM32, a member of the TRIM protein family, is a well-characterized E3 ubiquitin ligase. Co-IP experiments confirmed the interaction between PLAAT2 and TRIM32 (Fig. 5B). Western blot analysis showed that PLAAT2 knockdown or overexpression did not alter TRIM32 protein levels, indicating that PLAAT2 does not regulate TRIM32 expression (Fig. 5C). Next, we investigated whether TRIM32, as an E3 ubiquitin ligase, could regulate cMyc ubiquitination. Co-IP experiments confirmed the interaction between TRIM32 and cMyc, and IF demonstrated their co-localization (Fig. 5D, E). CHX chase assays revealed that TRIM32 knockdown slowed cMyc degradation, whereas TRIM32 overexpression accelerated it (Fig. 5F, G). Treatment with the proteasome inhibitor MG132 could reverse the effects of TRIM32 knockdown or overexpression on cMyc expression (Fig. 5H, I). Additionally, co-IP experiments showed that TRIM32 knockdown reduced cMyc ubiquitination, whereas TRIM32 enhanced it, further supporting the role of TRIM32 as an E3 ligase mediating cMyc ubiquitination (Fig. 5J, K).

### TRIM32 mediated K27- and K48-linked ubiquitination of cMyc at lysine residues 143 and 157

**Fig. 6:**
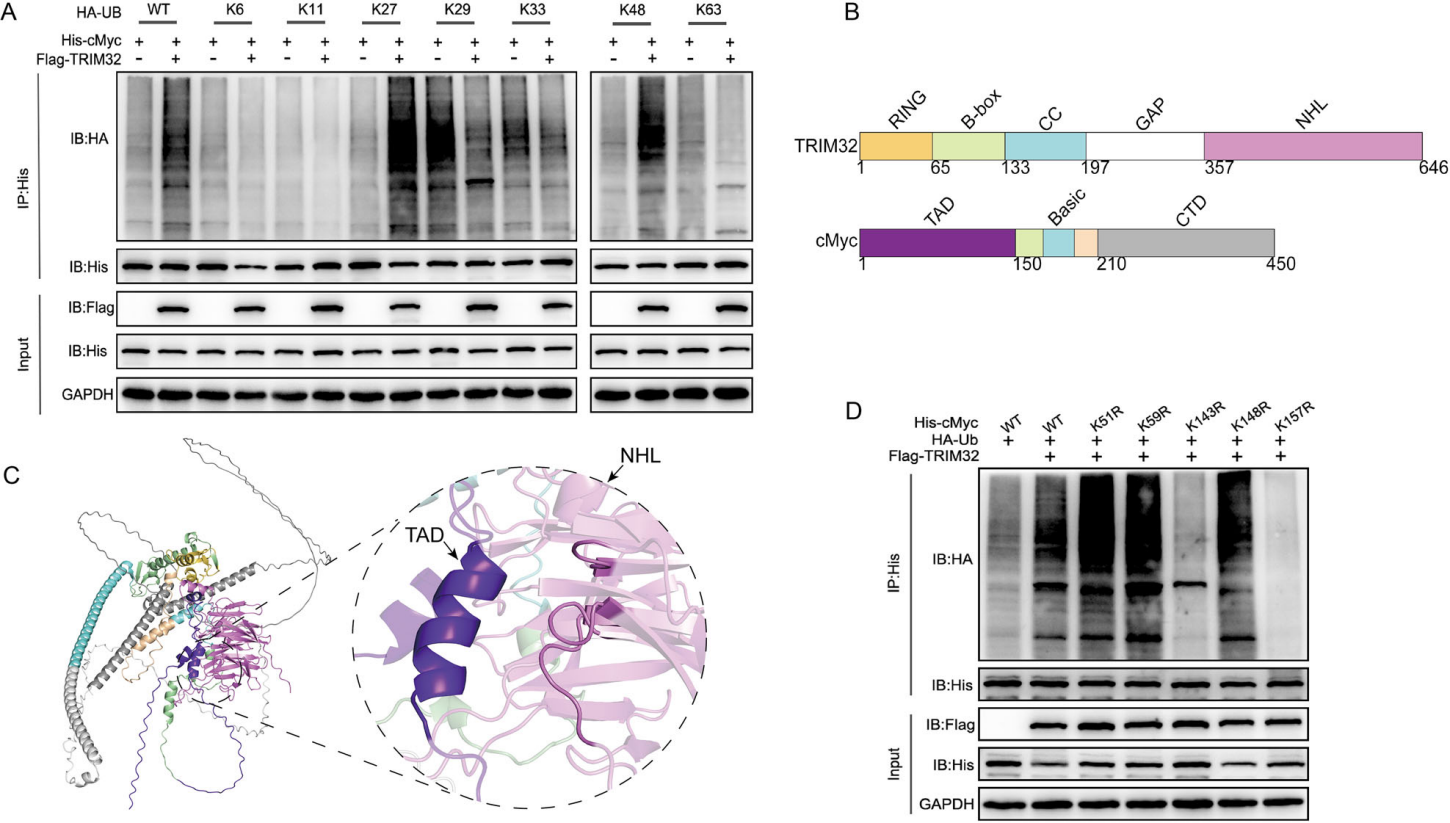
TRIM32 mediated K27 and K48-linked ubiquitination of cMyc at lysine residues 143 and 157. **A** Co-transfection of Flag-TRIM32, His-cMyc, HA-Ub wild-type, and mutant plasmids (Ub-K6, Ub-K11, Ub-K27, Ub-K29, Ub-K33, Ub-K48, and Ub-K63) in HEK293T cells, followed by Co-IP analysis of cMyc ubiquitination status. **B** NCBI analysis of TRIM32 and cMyc domain structures. **C** AlphaFold3 prediction of the 3D structures and interaction models of TRIM32 and cMyc. **D** Co-transfection of Flag-TRIM32, HA-Ub, and His-cMyc point mutation plasmids (K51R, K52R, K143R, K148R, and K157R) in HEK293T cells, followed by Co-IP analysis of cMyc ubiquitination levels.

We constructed wild-type ubiquitin overexpression plasmids and mutant plasmids retaining only one lysine residue (Ub-K6, Ub-K11, Ub-K27, Ub-K29, Ub-K33, Ub-K48, and Ub-K63) to further characterize the ubiquitination modifications mediated by TRIM32. These plasmids were co-transfected with Flag-TRIM32 and His-cMyc into HEK293T cells. After 24 h, the cells were treated with MG132 to prevent protein degradation. Co-IP experiments demonstrated that TRIM32 enhanced K27- and K48-linked ubiquitination of cMyc (Fig. 6A). We analyzed the domain sequences of TRIM32 and cMyc using the NCBI database to identify the specific lysine residues on cMyc targeted by TRIM32 (Fig. 6B) and predicted their three-dimensional structures and interaction models using AlphaFold3. The highest pTM-scored structure was selected for interaction analysis in PyMOL (version 2.5.6), revealing that the NHL domain of TRIM32 interacted with the TAD domain of cMyc (Fig. 6C). Based on this prediction, we performed site-directed mutagenesis of lysine residues within or adjacent to the TAD domain (K51R, K52R, K143R, K148R, and K157R). Co-IP experiments showed that mutations at K143R and K157R significantly reduced cMyc ubiquitination, whereas mutations at K51R, K52R, and K148R had no effect, indicating that TRIM32 ubiquitinates cMyc at lysine residues 143 and 157 (Fig. 6D).

### PLAAT2 inhibited GC cell proliferation, migration, and invasion through cMyc

**Fig. 7:**
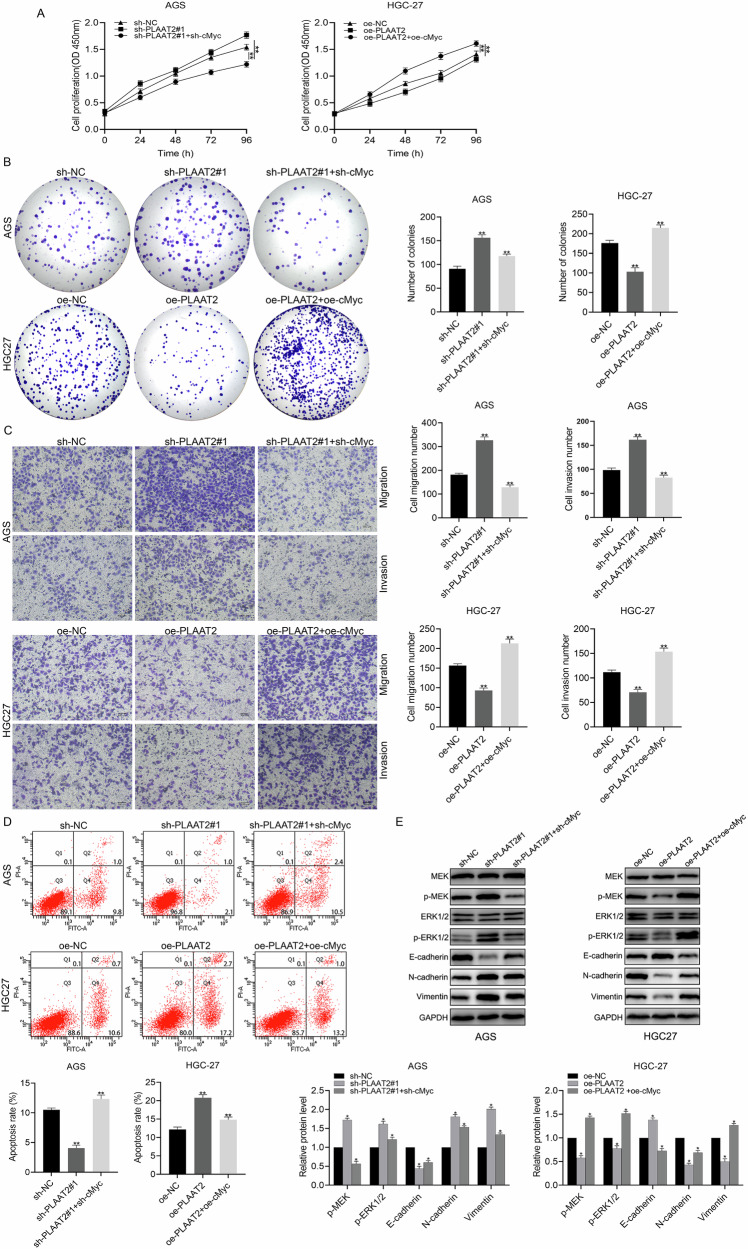
PLAAT2 inhibited the proliferation, migration, and invasion of gastric cancer cells through cMyc. **A** CCK-8 assay evaluating the effect of the PLAAT2/cMyc axis on gastric cancer cell proliferation. **B** Colony formation assay analyzing the impact of the PLAAT2/cMyc axis on clonogenic ability. **C** Transwell assay assessing changes in migration and invasion abilities mediated by the PLAAT2/cMyc axis. **D** Apoptosis assay analyzing the effect of the PLAAT2/cMyc axis on apoptosis levels. **E** Western blot analysis of the effect of the PLAAT2/cMyc axis on the MEK/ERK pathway and EMT-related proteins. ^*^P < 0.05, ^**^P < 0.01.

Previous studies have confirmed that PLAAT2 interacts with cMyc and suppresses its expression. We performed rescue experiments to further elucidate the role of the PLAAT2/cMyc axis in GC cell behavior. CCK-8 and colony formation assay results demonstrated that PLAAT2 knockdown enhanced GC cell proliferation, whereas simultaneous knockdown of PLAAT2 and cMyc reversed this effect. On the contrary, PLAAT2 overexpression inhibited proliferation, and simultaneous overexpression of cMyc reversed this inhibition (Fig. 7A, B). Transwell assays showed that cMyc knockdown or overexpression reversed the effects of PLAAT2 knockdown or overexpression on GC cell migration and invasion (Fig. 7C). Apoptosis assays revealed that PLAAT2 knockdown reduced apoptosis. In contrast, the simultaneous knockdown of PLAAT2 and cMyc restored apoptosis levels. PLAAT2 overexpression increased apoptosis, and simultaneous overexpression of cMyc reduced apoptosis (Fig. 7D). Western blot analysis demonstrated that PLAAT2 knockdown increased phosphorylated MEK and ERK1/2 levels and promoted EMT. Simultaneous knockdown of PLAAT2 and cMyc reversed these changes. In contrast, PLAAT2 overexpression decreased phosphorylated MEK and ERK1/2 levels, increased E-cadherin expression, and decreased N-cadherin and Vimentin expression. Simultaneous overexpression of PLAAT2 and cMyc also reversed these effects (Fig. 7E). These results indicated that PLAAT2 downregulated cMyc expression, inhibited EMT through the MEK/ERK signaling pathway, and ultimately suppressed GC progression.

### In vivo tumorigenesis assay validated the role of the PLAAT2/cMyc axis in suppressing GC progression

**Fig. 8:**
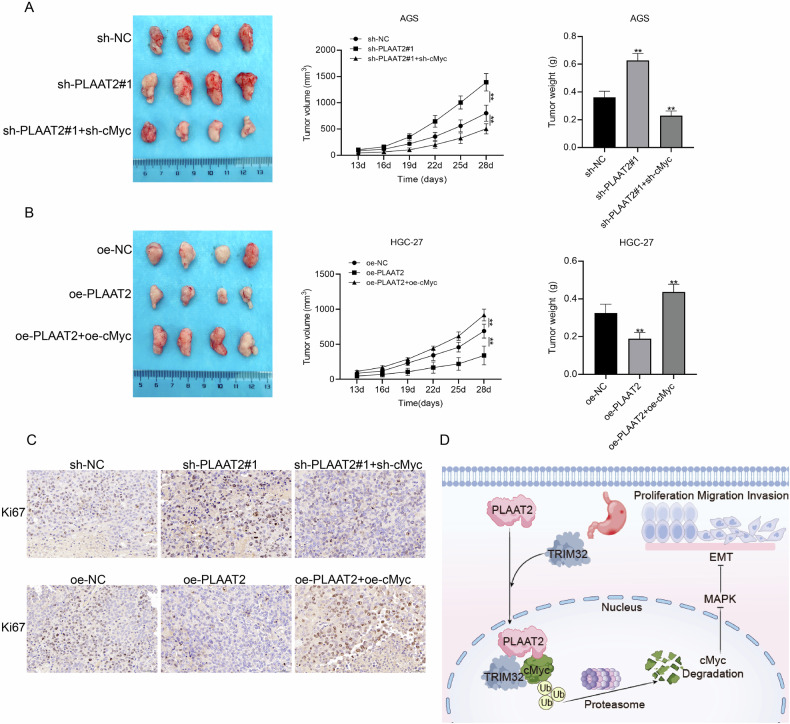
In vivo tumorigenesis assay validated the role of the PLAAT2/cMyc axis in suppressing malignant progression of gastric cancer. **A** Subcutaneous tumor formation assay in nude mice with simultaneous knockdown of PLAAT2 and cMyc, assessing tumor volume and weight changes. **B** Subcutaneous tumor formation assay in nude mice with simultaneous overexpression of PLAAT2 and cMyc, assessing tumor volume and weight changes. **C** Immunohistochemical analysis of Ki67 expression in nude mouse tumors. **D** Schematic diagram illustrating the working model of the study: PLAAT2 recruits TRIM32 to promote cMyc ubiquitination and degradation, leading to downregulation of cMyc expression, inhibition of EMT via the MEK/ERK signaling pathway, and regulation of gastric cancer proliferation, migration, and invasion. ^**^P < 0.01.

We established xenograft tumor models in nude mice to further investigate the role of PLAAT2 in vivo. The analysis of the tumor volume and weight revealed that PLAAT2 knockdown significantly promoted tumor growth, whereas simultaneous knockdown of cMyc reversed this effect (Fig. 8A). On the contrary, PLAAT2 overexpression inhibited tumor growth, and simultaneous overexpression of cMyc reversed this inhibition (Fig. 8B). These trends were consistent with the changes in Ki67 expression in tumor tissues (Fig. 8C). Together, these findings demonstrated that PLAAT2 inhibited GC progression in vivo by downregulating cMyc expression and suppressing malignant phenotypes (Fig. 8D).

## Discussion

GC is highly prevalent worldwide, with a significant number of cases occurring in East Asia. Moreover, patients with advanced GC often face a poor prognosis due to the high likelihood of metastasis and recurrence [19]. Therefore, further investigation into the molecular mechanisms driving GC development and progression may uncover novel therapeutic targets and improve clinical outcomes. PLAAT2, a member of the PLAAT family, comprises three domains: an N-terminal domain, a lecithin retinol acyltransferase (LRAT) domain, and a C-terminal domain. The LRAT domain catalyzes amino acid acylation, whereas the C-terminal domain plays a crucial role in supporting the growth of RAS-dependent cancer cells [20]. In our previous study, we demonstrated that the upstream promoter region of PLAAT2 was significantly enriched with CpG islands and its expression was negatively correlated with DNA methylation. Moreover, both PLAAT2 expression and promoter methylation status were closely associated with the prognosis of patients with GC [17]. Through further experiments, we confirmed that PLAAT2 was directly regulated by DNA methylation. Studies indicate that PLAAT2 can catalyze the conversion of phosphatidic acid into lysophosphatidic acid (LPA) and free fatty acids. LPA, a bioactive lipid, plays a key role in activating multiple tumor-associated signaling pathways, such as ERK [21], Yes-associated protein [22], MAPK [23], and phospholipase C/diacylglycerol kinase [24] signaling pathways. PLAAT2 is downregulated in colon, cervical, and breast cancers and can inhibit cancer cell proliferation [25]. The present study found that PLAAT2 was downregulated in GC, functioning as a tumor suppressor that influenced disease progression.

Tumor invasion and metastasis, which are intricate, multistep processes, primarily include local invasion, intravasation into blood and lymphatic vessels, and extravasation, wherein tumor cells leave the primary tumor site to form secondary tumors in adjacent or distant organs, leading to cancer-related mortality [26]. EMT is a hallmark event in tumor progression in which epithelial cells transform into mesenchymal-like cells. This enhances their invasiveness and resistance to treatments such as chemotherapy and targeted therapy, thereby reducing the efficacy of standard cancer therapies [27, 28]. EMT is associated with various malignant behaviors of tumors, including proliferation, invasion, and metastasis [29]. During EMT, cells lose adhesion to neighboring cells and the extracellular matrix, acquiring mesenchymal traits such as increased motility and invasiveness, thus enabling detachment from the primary tumor and spread to distant sites [30]. The phenotypic shift of epithelial cells to nonpolarized mesenchymal cells is a crucial step in EMT. The regulation of EMT involves multiple signaling pathways and regulatory factors [31, 32]. Among these, the MEK/ERK signaling pathway is pivotal in promoting GC progression and metastasis by modulating EMT [33]. Stanniocalcin 2 (STC2) overexpression promotes cell proliferation and migration in colon cancer, accompanied by the upregulation of key signaling molecules including RAS, p-MEK, and p-ERK, along with decreased E-cadherin and increased N-cadherin expression. These findings suggest that STC2 facilitates EMT and drives colon cancer progression by activating the MEK/ERK signaling pathway [34]. Methionine sulfoxide reductase B1 (MsrB1) is highly expressed and correlates with postoperative prognosis in liver cancer. The knockdown of MsrB1 suppresses the migration and invasion of HCCLM3 cells through inhibiting EMT. Additionally, the phosphorylation of key MAPK pathway components, including MEK, ERK1/2, and P53, is reduced following MsrB1 silencing [35]. The overexpression of nuclear factor I C1 suppresses the malignant potential of MDA-MB-231 cells, increases E-cadherin expression, and decreases N-cadherin expression in breast cancer, while reducing the expression of p-MEK and p-ERK [36]. However, the link between PLAAT2 and EMT remains poorly defined. GSEA indicated a possible association between PLAAT2 and the MAPK pathway, suggesting a role of PLAAT2 in EMT regulation. In this study, the knockdown of PLAAT2 did not affect total MEK and ERK1/2 levels but significantly increased p-MEK and p-ERK1/2 expression, reduced E-cadherin expression, and elevated N-cadherin and Vimentin expression. In contrast, PLAAT2 overexpression decreased the phosphorylation of MEK and ERK1/2 while reversing EMT marker expression, without altering total MEK and ERK1/2 levels. These results suggested that PLAAT2 might inhibit EMT by modulating the MEK/ERK pathway, thereby limiting GC cell proliferation, migration, and invasion.

Accumulating evidence indicates a significant contribution of epigenetic mechanisms to GC initiation and progression [37]. Ubiquitination, a crucial epigenetic posttranslational modification, regulates protein stability and significantly influences tumorigenesis by affecting cancer cell proliferation, apoptosis, and metastasis [38]. In this study, we used IP-MS, co-IP, and IF assays and found that PLAAT2 interacted and co-localized with cMyc and promoted its degradation via the ubiquitin–proteasome pathway. Further analysis revealed that PLAAT2 recruited TRIM32 to mediate K27- and K48-linked ubiquitination of cMyc at lysine residues 143 and 157. cMyc is broadly expressed across various tissues and organs, particularly during early embryonic development. Its dysregulation is frequently observed in aggressive tumors and often associated with poor patient outcomes [39]. Abnormal cMyc expression can be triggered in tumor cells by gene amplification, chromosomal rearrangements, single nucleotide polymorphisms in regulatory regions, mutations in signaling pathways, or ubiquitin-dependent protein degradation pathways [40, 41]. Studies have shown that ubiquitin-specific peptidase 1 (USP1) acts as an oncogene in bladder cancer by stabilizing cMyc through deubiquitination, thus promoting cancer progression [42]. A disintegrin and metalloproteinase with thrombospondin motifs 4 stabilizes cMyc in lung cancer by inhibiting its ubiquitination, activating the MAPK signaling pathway, and promoting lung cancer progression [43]. TRIM55 directly interacts with cMyc in colon cancer, promoting its ubiquitination and downregulating its expression, thereby inhibiting colon cancer development [44]. Studies have shown high expression of cMyc in liver cancer. USP19 directly binds to cMyc, stabilizing it via deubiquitination, thereby regulating the malignant behavior of liver cancer cells [45]. However, our study had certain limitations. Although we established a mouse xenograft tumor model, it did not fully recapitulate the complexity of the human tumor microenvironment. In future studies, we plan to employ patient-derived xenograft models or genetically engineered mouse models to more accurately mimic the biological characteristics of GC. Collectively, our findings suggested that PLAAT2 acted as a tumor suppressor in GC by promoting cMyc degradation and inhibiting MEK/ERK-mediated EMT, ultimately limiting tumor progression.

## Conclusions

PLAAT2 is downregulated in GC, and functional assays confirm its tumor-suppressive role. Mechanistically, PLAAT2 recruits the E3 ligase TRIM32, which then promotes cMyc ubiquitination and degradation, ultimately leading to the inactivation of the MEK/ERK pathway. These findings suggest that PLAAT2 functions as a crucial negative regulator of GC progression and a promising therapeutic target.

## Supplementary information

Supplementary Material

Original image for checking

## Data Availability

The raw data of this study are derived from the TCGA database (https://portal.gdc.cancer.gov/) and GEO database (https://www.ncbi.nlm.nih.gov/geo/) which are publicly available databases.
