# Supplementary material for: PLAAT2 suppresses gastric cancer progression by facilitating cMyc ubiquitination and inhibiting MEK/ERK signaling
Source: Cell Death Dis. 2026 Mar 18;17(1):314. doi: 10.1038/s41419-026-08546-y (PMC13039291; doi:10.1038/s41419-026-08546-y)
Supplement: Supplementary file 1 — Supplementary Material [file 41419_2026_8546_MOESM1_ESM.docx]

**Supplementary Figure legends:**

**Supplementary Fig. 1** **PLAAT2 expression is regulated by DNA methylation**. (A) The online prediction website MethPrimer was utilized to analyze the promoter region sequence of PLAAT2, revealing the presence of a CpG island. (B) Distribution of PLAAT2 promoter methylation in TCGA gastric cancer samples with fitted normal curve, the short black bar indicates the methylation value in noncancerous tissues. (C) The expression of PLAAT2 is negatively correlated with its methylation level. (D) The hypermethylation group of PLAAT2 showed a worse prognosis compared to the hypomethylation group. (E) The promoter region of PLAAT2 is hypomethylated in the normal gastric mucosal cell line GES-1 but hypermethylated in five gastric cancer cell lines. (F) After treatment with the demethylating drug 5-Aza-CdR, the expression of PLAAT2 increased at the protein level, and this effect was dose-dependent.

**
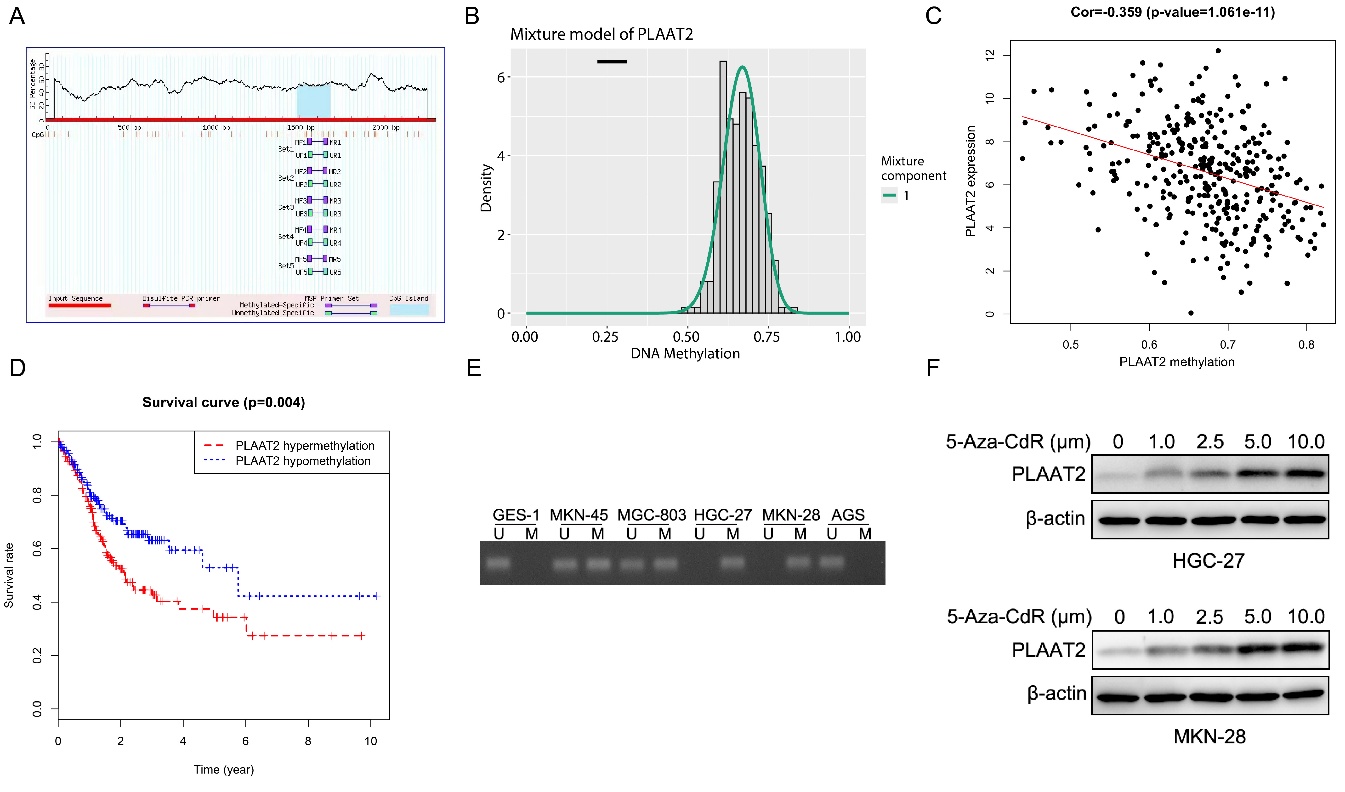
**


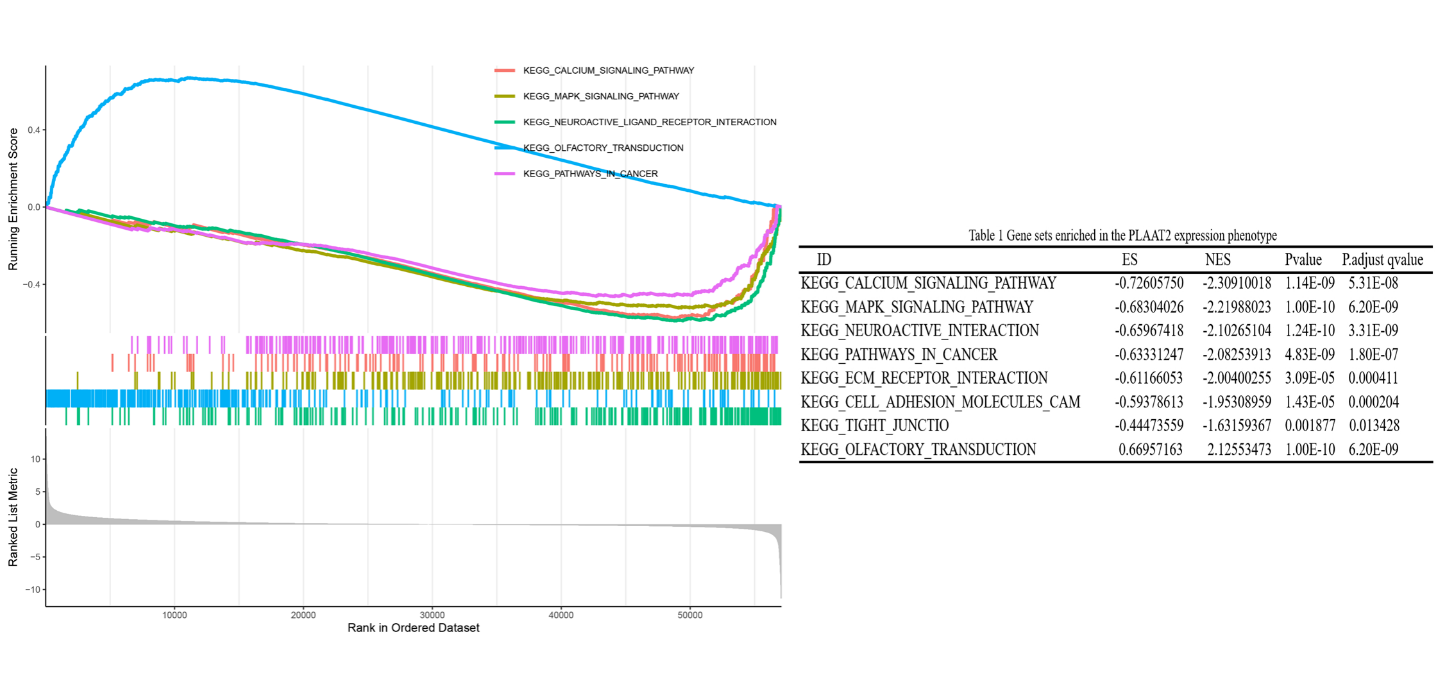
**Supplementary Fig. 2 Enrichment analysis of single gene PLAAT2 based on TCGA database**. PLAAT2 is most commonly found in the MAPK pathway, extracellular matrix (ECM) receptor interactions, cell adhesion molecules (CAM), and cancer-related signaling pathways.

Supplementary Table 1. The sequences of shRNAs are shown in this study

shRNA-name Sequence (5' −> 3')

sh-NC sense UUCUCCGAACGUGUCACGUTT

anti-sense ACGUGACACGUUCGGAGAATT

sh-PLAAT2 #1 sense GACAACUACAGGGUCAAUATT

anti-sense UAUUGACCCUGUAGUUGUCTT

sh-PLAAT2 #2 sense GCGAGCACUUCGUGAACCATT

anti-sense UGGUUCACGAAGUGCUCGCTT

sh-cMyc sense GCCGUAUUUCUACUGCGACTT

anti-sense GUCGCAGUAGAAAUACGGCTT

sh-TRIM32 sense GACUUUGGAGAGAAGUUAATT

anti-sense UUAACUUCUCUCCAAAGUCTT

sh-GAPDH sense UGACCUCAACUACAUGGUUTT

anti-sense AACCAUGUAGUUGAGGUCATT

Supplementary Table 2. The primers used in this study

Gene name Sequence (5' −> 3')

PLAAT2 Forward Primer AGACCGAGACTTGGAGACCTGATTG

Reverse Primer TTGTTGGAAGGCAGTGGTGTGTATC

cMyc Forward Primer CAGAGGAGCAAAAGCTCATTTTC

Reverse Primer CAAGAGTTCCGTAGCTGTTCAAG

TRIM32 Forward Primer GCTCCTTAAGGTAGGTCATGTTG

Reverse Primer AAGTAACAGAGGTAGAGGCAGCA

GAPDH Forward Primer CAAGGCTGTGGGCAAGGTCATC

Reverse Primer GTGTCGCTGTTGAAGTCAGAGGAG

Supplementary Table 3. The relationship between PLAAT2 expression and clinical pathological characteristics of gastric cancer patients

| Clinical characteristics | PLAAT2 expression | | Cases | *P* value |
| --- | --- | --- | --- | --- |
|  | Positive | Negative |  |  |
| Gender | 32 | 84 |  | 0.420 |
| Male | 17（0.53） | 47（0.56） | 64 |  |
| Female | 15（0.47） | 37（0.44） | 52 |  |
| Age,year |  |  |  | 0.322 |
| ＜60 | 21（0.66） | 41（0.49） | 62 |  |
| ≥60 | 11（0.34） | 43（0.51） | 54 |  |
| Tumor size,cm |  |  |  | 0.306 |
| ＜5 | 7（0.22） | 44（0.52） | 51 |  |
| ≥5 | 25（0.78） | 40（0.48） | 65 |  |
| T stage |  |  |  | 0.013* |
| T1+T2 | 20（0.62） | 36（0.43） | 56 |  |
| T3+T4 | 12（0.38） | 48（0.57） | 60 |  |
| Lymph node metastasis |  |  |  | 0.020* |
| No | 18（0.56） | 39（0.46） | 57 |  |
| Yes | 14（0.44） | 45（0.54） | 59 |  |
| TNM stage |  |  |  | 0.005* |
| Ⅰ+Ⅱ | 10（0.31） | 30（0.36） | 40 |  |
| Ⅲ | 22（0.69） | 54（0.64） | 76 |  |

* means *P*＜0.05

Supplementary Table 4. The MSP primers used in this study

Gene name Sequence (5' −> 3')

PLAAT2 M-Forward Primer AGTATTATAGTTTTTAAGGTGGCGC

M-Reverse Primer TACAATTTCACTCCTAAACCAACG

U-Forward Primer TATTATAGTTTTTAAGGTGGTGTGT

U-Reverse Primer TACAATTTCACTCCTAAACCAACAA

**Reagents and antibodies**

The following antibodies were used: anti-PLAAT2 (1:2000, Thermo, cat# PA5-50680), anti p-MEK (1:2000, Proteintech, cat# 28930-1-AP), anti-MEK (1:5000, Proteintech, cat# 11049-1-AP), anti p-ERK1/2 (1:2000, Proteintech, cat# 11257-1-AP), anti-ERK1/2 (1:2000, Proteintech, cat# 28733-1-AP), anti-E-cadherin (1:5000, Proteintech, cat# 20874-1-AP), anti-N-cadherin (1:5000, Proteintech, cat# 22018-1-AP), anti-Vimentin (1:5000, Proteintech, cat# 10366-1-AP), anti-cMyc (1:2000, Proteintech, cat# 10828-1-AP), anti-TRIM32 (1:5000, Proteintech, cat# 10326-1-AP), anti-Ubiquitin (1:1000, Proteintech, cat# 10201-2-AP), anti-GAPDH (1:10000, Proteintech, cat# 10494-1-AP), and anti-β-actin (1:10000, Proteintech, cat# 20536-1-AP), anti-Flag (1:2000, Proteintech, cat# 20543-1-AP), anti-His (1:1000, Proteintech, cat# 10001-0-AP).
