# Supplementary material for: PLAAT2 suppresses gastric cancer progression by facilitating cMyc ubiquitination and inhibiting MEK/ERK signaling
Source: Cell Death Dis. 2026 Mar 18;17(1):314. doi: 10.1038/s41419-026-08546-y (PMC13039291; doi:10.1038/s41419-026-08546-y)
Supplement: Supplementary file 2 — Original image for checking [file 41419_2026_8546_MOESM2_ESM.docx]

Fig.1 G







β-actin β-actin







PLAAT2 PLAAT2





β-actin


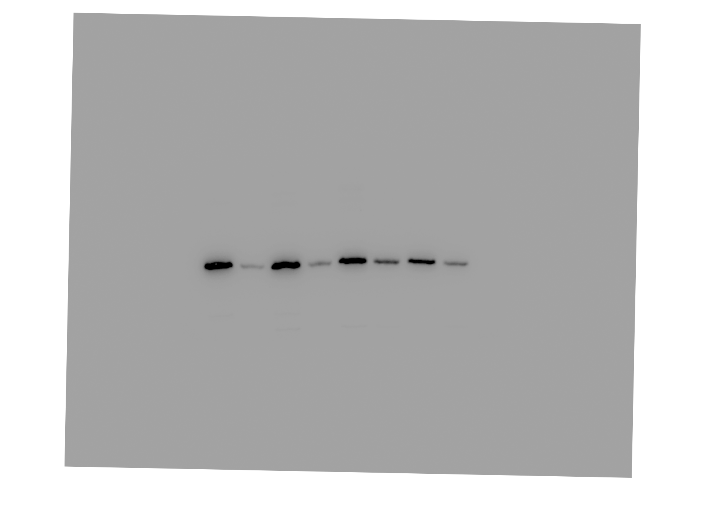


PLAAT2



Fig.1 I

β-actin





PLAAT2





Fig.2 A

β-actin β-actin







PLAAT2 PLAAT2

AGS MGC803

Fig.2 F











MEK p-MEK MEK p-MEK











ERK p-ERK ERK p-ERK










E-cadherin N-cadherin E-cadherin N-cadherin











Vimentin GAPDH Vimentin GAPDH

AGS MGC803





Fig.3 A

β-actin β-actin







PLAAT2 PLAAT2

HGC27 MKN28

Fig.3 F











MEK p-MEK MEK p-MEK











ERK p-ERK ERK p-ERK












E-cadherin N-cadherin E-cadherin N-cadherin











Vimentin GAPDH Vimentin GAPDH

HGC27 MKN28

Fig.4 B











PLAAT2 cMyc PLAAT2 cMyc











cMyc PLAAT2 cMyc PLAAT2

AGS HGC27

Fig.4 D







PLAAT2 PLAAT2







β-actin β-actin







cMyc cMyc







β-actin β-actin

AGS HGC27

Fig.4 F Fig.4 G











PLAAT2 PLAAT2











GAPDH GAPDH

AGS HGC27

Fig.4 H Fig.4 I







PLAAT2 PLAAT2








 cMyc cMyc





GAPDH GAPDH

AGS HGC27





Fig.4 J

PLAAT2 cMyc







GAPDH Ub

AGS

Fig.4 K







PLAAT2 cMyc







GAPDH Ub

HGC27

Fig5 B











PLAAT2 TRIM32 PLAAT2 TRIM32











TRIM32 PLAAT2 TRIM32 PLAAT2

AGS HGC27

Fig5 C







PLAAT2







TRIM32







GAPDH

AGS HGC27

Fig5 D

TRIM32 cMyc TRIM32 cMyc

cMyc TRIM32 cMyc TRIM32

AGS HGC27

Fig5 F Fig5 G

cMyc cMyc

GAPDH GAPDH

AGS HGC27

Fig5 H Fig5 I

TRIM32 TRIM32

cMyc cMyc

GAPDH GAPDH

AGS HGC27

Fig5 J

cMyc TRIM32

GAPDH Ub

AGS

Fig5 K

CMyc TRIM32

GAPDH Ub

HGC27

Fig6 A

Ub

cMyc

TRIM32

cMyc

GAPDH

Fig6 D

Ub cMyc

TRIM32 cMyc

GAPDH

Fig7 E

MEK p-MEK

ERK p-ERK

E-cadherin N-cadherin

Vimentin GAPDH

AGS

MEK p-MEK

ERK p-ERK

E-cadherin N-cadherin

Vimentin GAPDH

HGC27

Supplementary Fig1 F

PLAAT2 β-actin

HGC-27

PLAAT2 β-actin

MKN-28
